# In-SEM micro-machining reveals the origins of the size effect in the cutting energy

**DOI:** 10.1038/s41598-021-81125-7

**Published:** 2021-01-22

**Authors:** Bentejui Medina-Clavijo, Gorka Ortiz-de-Zarate, Andres Sela, Iñaki M. Arrieta, Aleksandr Fedorets, Pedro J. Arrazola, Andrey Chuvilin

**Affiliations:** 1 Electron-Microscopy Laboratory, CIC nanoGUNE BRTA, 20018 Donostia, Spain; 2 Faculty of Engineering, Mondragon Unibertsitatea, 20500 Arrasate, Spain; 3 Far Eastern Federal University, Vladivostok, 690950 Russia; 4 Basque Foundation for Science, 48013 Bilbao, Spain

**Keywords:** Structural materials, Mechanical engineering, Characterization and analytical techniques, Imaging techniques, Microscopy

## Abstract

High-precision metal cutting is increasingly relevant in advanced applications. Such precision normally requires a cutting feed in the micron or even sub-micron dimension scale, which raises questions about applicability of concepts developed in industrial scale machining. To address this challenge, we have developed a device to perform linear cutting with force measurement in the vacuum chamber of an electron microscope, which has been utilised to study the cutting process down to 200 nm of the feed and the tool tip radius. The machining experiments carried out in-operando in SEM have shown that the main classical deformation zones of metal cutting: primary, secondary and tertiary shear zones—were preserved even at sub-micron feeds. In-operando observations and subsequent structural analysis in FIB/SEM revealed a number of microstructural peculiarities, such as: a substantial increase of the cutting force related to the development of the primary shear zone; dependence of the ternary shear zone thickness on the underlaying grain crystal orientation. Measurement of the cutting forces at deep submicron feeds and cutting tool apex radii has been exploited to discriminate different sources for the size effect on the cutting energy (dependence of the energy on the feed and tool radius). It was observed that typical industrial values of feed and tool radius imposes a size effect determined primarily by geometrical factors, while in a sub-micrometre feed range the contribution of the strain hardening in the primary share zone becomes relevant.

## Introduction

Machining is a high added value process, representing approximately 5% of the GDP in developed countries. Gaining a deeper understanding of the physical mechanisms which underpin metal cutting will help to reduce manufacturing costs and improve component quality. Interaction of the cutting tool with the workpiece imposes enormous deformation work in a small volume in a very short time, which complicates the study of the thermomechanical processes at the cutting edge by conventional means. Up till now this challenge has been addressed by post-machining structural analysis of the tool, workpiece^1^ and the chip^2^, different theoretical approaches summarized in^3–5^, or fast rate in-operando optical analysis utilizing high speed cameras^6–9^.

Structural features of metals, significant for cutting, have scales down to sub-100 nm and are assessed by different electron microscopy techniques. Thermomechanical processes, defining plasticity and thus governing cutting, also have characteristic length scales well below one micron. In an effort to access and visualise machining operations at the micro-/nano-scale, cutting devices have been developed inside Scanning Electron Microscopes (SEM). The first device of this kind was reported by a Japanese group in a series of works starting from 1989^10^. Later, Heo (2004) has reported a linear micro-machining setup inside an SEM chamber^11,12^ able to control feed in the range of tens of microns. The process of machining of tungsten carbide by a polycrystalline diamond tool was studied and observations of cutting progress were made by taking the still images after stopping the process every 100 µm. In his works Heo reported forces in the range of tens of Newtons measured in-situ^11^, however, no description of how this was done was therein reported. Recently Fang has developed an in-SEM linear cutting device with ultra-high precision positioning^13^, which allowed feed values down to 10 nm. This allowed the observation of a ductile to brittle cutting transition in monocrystalline silicon cut by a diamond tool. These few works, to the best of our knowledge, represent all approaches to in-situ SEM machining made to date. The limited progress in this area is due to the complexity of incorporating micro-devices inside an SEM chamber, and limited availability of this kind of equipment within the metal processing community.

The advantage of in-SEM machining lies in the ability to perform a deep microstructural analysis of the cutting process based on a variety of spectroscopic and structural techniques provided by SEM. Cutting, in this case, is carried out in a vacuum chamber that prevents oxidation of microstructural features produced during cutting. Besides structural and compositional data, measurement of the forces involved in cutting is an even more important source of information because it relates to the energetics of the process. Such measurements have not been realised so far in micro-cutting devices on the scales of sub-micrometre feeds and micro-Newton forces.

A comparative study of forces in the machining process at different scales requires an invariant to the cutting dimensions. A commonly accepted size-invariant parameter for multiscale studies is the so called specific cutting energy (*K*_*c*_). This quantity is a measure of the energy needed for cutting a unitary volume of material and has a specific standard value for each material^14^. In an ideal case, *K*_*c*_ would be independent of the cutting parameters.

In reality, it has been experimentally observed that the *K*_*c*_ value monotonically increases with the decrease of the uncut chip thickness (*h*)^14,15^. Two main hypotheses were proposed to explain this phenomenon. One considers that the relative contribution of tool-workpiece friction increases while *h* decreases^16^, which leads to the higher values of the specific cutting energy at low feeds. This effect becomes relevant when *h* decreases to values close or below the tool edge radius. The latter is quantified by the relative tool sharpness (RTS), the ratio between *h* and the tool edge radius^17^. Several works have successfully described *K*_*c*_ as being solely a function of RTS, meaning it is a purely geometrical effect^18,19^. In contrast to this approach, it has been suggested that the material strengthening due to higher strain concentration^20^ at small scales can contribute to the specific energy increase^21,22^ at small tool radii. This suggestion is based on a discrete mechanics of solids, where a major increase in dislocation density causes material hardening. These two hypotheses introduce different dependencies of the *K*_*c*_—in the first case it depends only on RTS, in the second case there should also be an independent contribution of the tool edge radius. Though several studies have been made to resolve this ambiguity between both hypotheses, a limited range of *h* and tool radii accessible in macroscopic machining has not allowed for a definite explanation so far. Understanding the underlaying mechanisms of this size effect may be important for optimisation of the cutting conditions and the tool geometry in practical applications.

In the present work the concept of a versatile micro-cutting in-SEM setup is developed, a prototypic device is constructed, and proof of concept experiments are performed. On top of structural and compositional analysis of the components involved in the cutting process, this prototype allows measurement of the cutting force. These microscopic measurements in combination with macroscopic cutting experiments of the same material (annealed aluminium 7475) enable the study of size dependence of the specific cutting energy in a range of tool radii varying by two orders of magnitude. A simple model, quantitatively describing the behaviour of the cutting energy in the whole range of employed cutting parameters, is proposed and validated.

## Results

### Multi-scale cutting: in-SEM and macroscopic cutting setups

**Figure 1:**
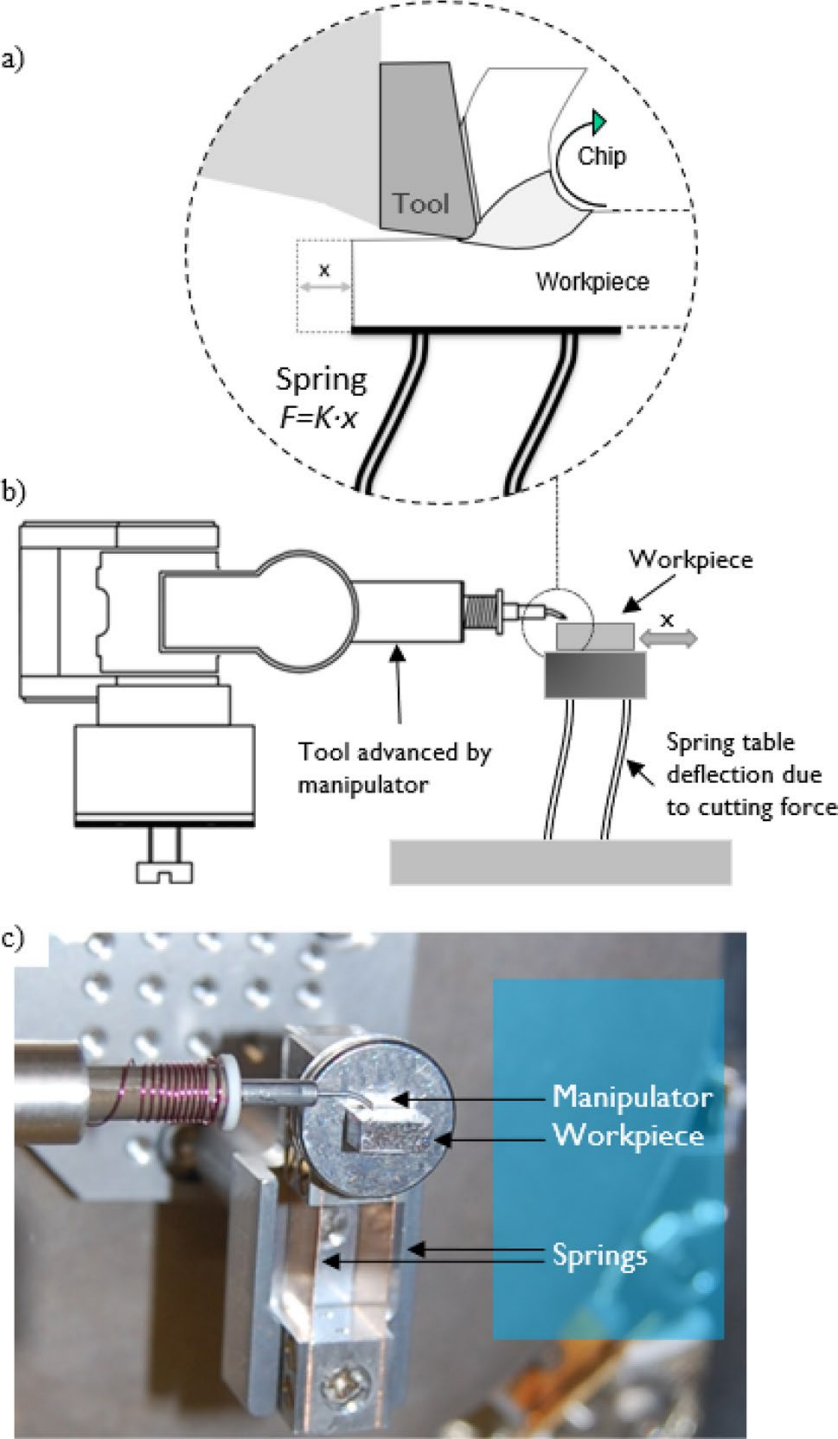
(**a**) Sketch of the working principle of force measurement; (**b**) schematics of an actual implementation of the micro-cutting system with the force measurement; (**c**) photo of a real system inside FIB/SEM chamber.

This work is enabled by microscopic and macroscopic machining experiments. For the microscopic experiments a device for linear micro-machining has been designed and build ad-hoc for this work. The design of this in-SEM cutting device with force measurement is described in detail in “Methods” section. In brief (see also Fig. 1): the workpiece is fixed on a spring with a calibrated force constant (Kleindiek Nanotechnik GmbH STFMA spring-table system); FIB nanofabricated WC–Co tool is fixed on the tip of the arm of a piezoelectric nanomanipulator (Kleindiek Nanotechnik GmbH MM3A-EM); two out of three axis of the MM3A are used to feed and advance the tool against the workpiece; the movement of tool and deflection of the workpiece is monitored via SEM (FEI Co. Helios 600 Nanolab DualBeam) imaging; the deflection of the sample is used to measure the cutting force via a spring force constant.

Macroscopic cutting experiments were performed on a linear bench (High Speed Machining Centre Lagun) with a dynamometer (TPUN 160308 TTM—uncoated), see “Methods” section for detailed description. Very small uncut chip thicknesses were precisely measured a posteriori by Alicona IFG4 profilometer.

All experiments were performed on the same annealed and rolled Aluminium 7475 material.

### Features of micro-cutting process

**Figure 2:**
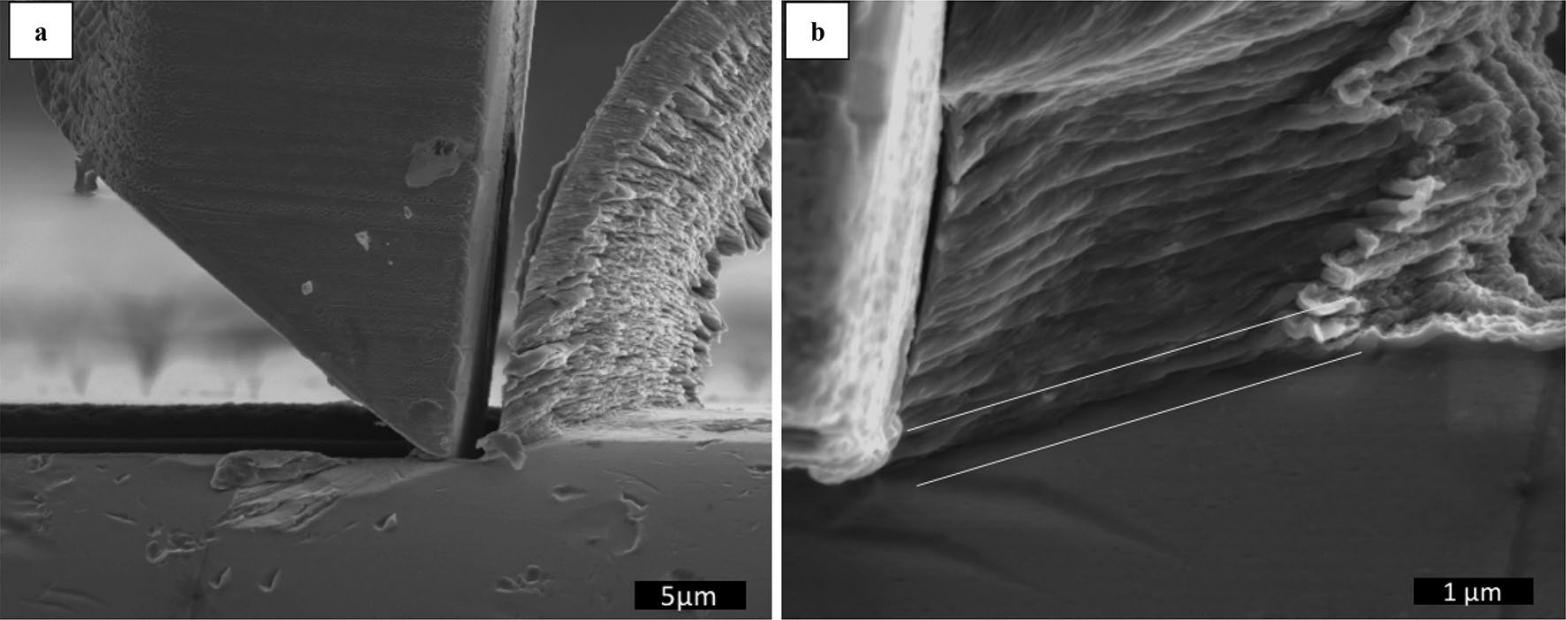
(**a**) Lateral overview of the system workpiece-chip-tool. (**b**) Steady state shape of fully developed PSZ, PSZ is marked by white lines.

The experiments of in-situ cutting show all features typical for an orthogonal cutting process. While the tool advances against the workpiece, the chip is constantly generated by a shear from the tool apex to the workpiece surface in a similar fashion to the classical Merchant’s description of the primary shear zone^23^, as shown in Fig. 2a,b. Figure 2b depicts a side view near the tool tip region during a quick stop in a cut with *h* = 1.18 µm at 0.6 mm/min cutting speed. After the PSZ is fully developed and a chip is being generated, the chip thickness here is ~ 4 µm. Development of the PSZ means that the material deformation is concentrated in a narrow region around a shear line (see white marks on Fig. 2b).

**Figure 3:**
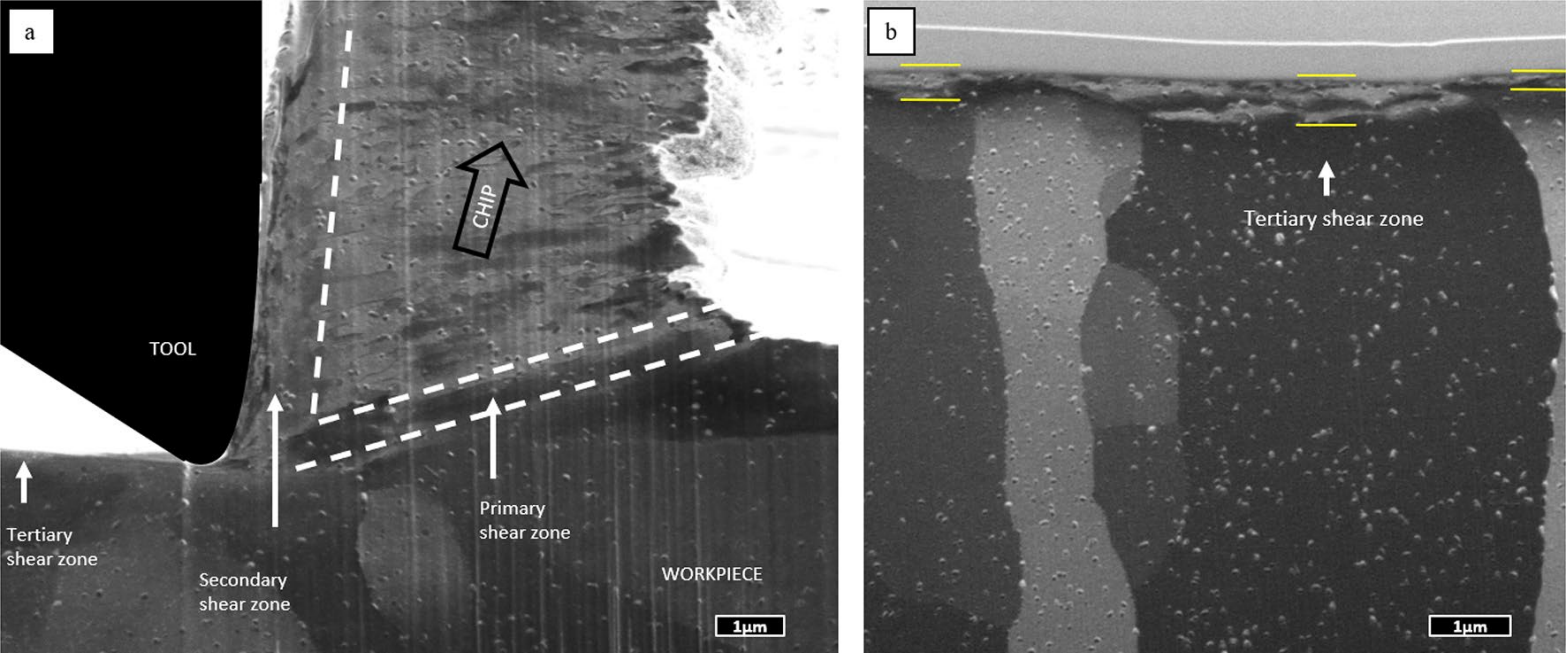
(**a**) Cross-section of a chip on a workpiece cut with 1 µm feed and 0.6 mm/min imaged by channelling contrast in FIB. (**b**) Detail of the size of tertiary shear zone after cutting action on grains with different orientation.

Besides on-line SEM monitoring of the micro-cutting process, the microstructure generated during cutting experiments was a posteriori analysed by FIB and SEM. For this purpose, the cross-sections of the samples after the cuts were prepared by ion milling. The final chip residing on the workpiece was sectioned along the cut together with the workpiece at the half of the cutting width, i.e. the cross-section represents the structure in the middle of the cut. The grain structure in the cross-section was accessed by FIB imaging exploiting the very high channelling contrast obtainable in ion beam scanning^24^. Figure 3a shows an example of thus obtained cross-section prepared after a final quick-stop.

Variations in channelling contrast in Fig. 3a reflect different crystallographic orientations of the crystal grains, revealing canonical zones known in cutting theory. The workpiece is characterised by large (~ 10 μm) crystal grains with uniformly distributed Guinier–Preston zones^25^, a typical product of age hardening in aeronautic aluminium. The chip consists of elongated highly deformed crystal grains oriented along the PSZ. The PSZ is visible as a region where the structure of the workpiece changes to that of the chips. The Secondary shear zone (indicated by an arrow on Fig. 3a), resulting from the friction of the chip over the tool, is characterised by a nanocrystalline material notoriously different in grain size and orientation to the rest of the chip. And finally, a sub-micrometre layer of deformed material on the surface of the workpiece after the cutting tool, the TSZ, develops in different manner depending on the orientation of the grain been cut. Figure 3b shows this region in more detail: Several grains with different orientation (evidenced by substantially different channelling contrast) develop TSZ, which differs in thickness by a factor of 4 (0.2 to 0.8 μm).

### Cutting force in micro- and macro machining

**Figure 4:**
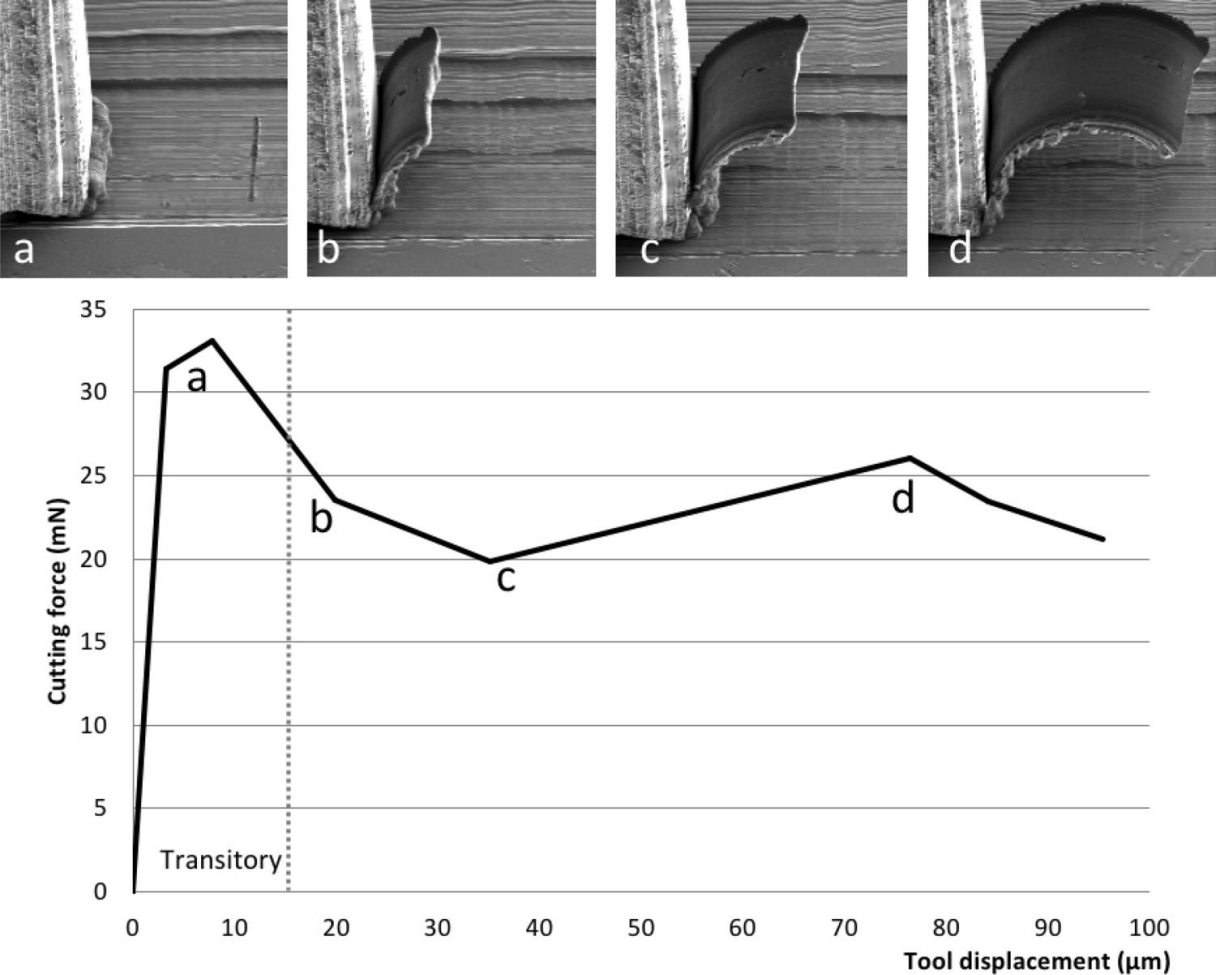
Representation of cutting force vs. cutting displacement.

Cutting force measurements in micro-cutting experiments were made by tracking the workpiece displacement on SEM images as described in methods section. Figure 4 shows a typical image sequence of the cutting experiment and a corresponding force vs. tool displacement curve. The example in Fig. 4 is obtained by cutting with an uncut chip thickness (*h*) of 1.1 µm, and width of 12 µm. A characteristic feature of the curve is a substantially higher force at the beginning of cutting. A subsequent steady state after ~ 20 μm of cutting shows values which are 20–30% lower than the initial force.

This initial force increase may be attributed to a stick–slip movement or could correspond to the development of the PSZ, when the deformation is still not concentrated in the band of shearing. From here on, for further force comparisons and discussion about specific cutting force (cutting energy) the values of force in steady state will be used, i.e. in this example a mean value of points b,c,d and assuming an error of ± 10%.

For the macroscopic case, the values of the cutting force were obtained by a force sensor. Three different edge radii (24, 11 and 5 µm) were used. For a proper comparison of the values of forces obtained in in-situ SEM and in the machining bench, the cutting forces were normalized to the cutting width in all the cases, which is in the order of micrometres for in-situ cutting and one millimetre for the machining bench.

**Figure 5:**
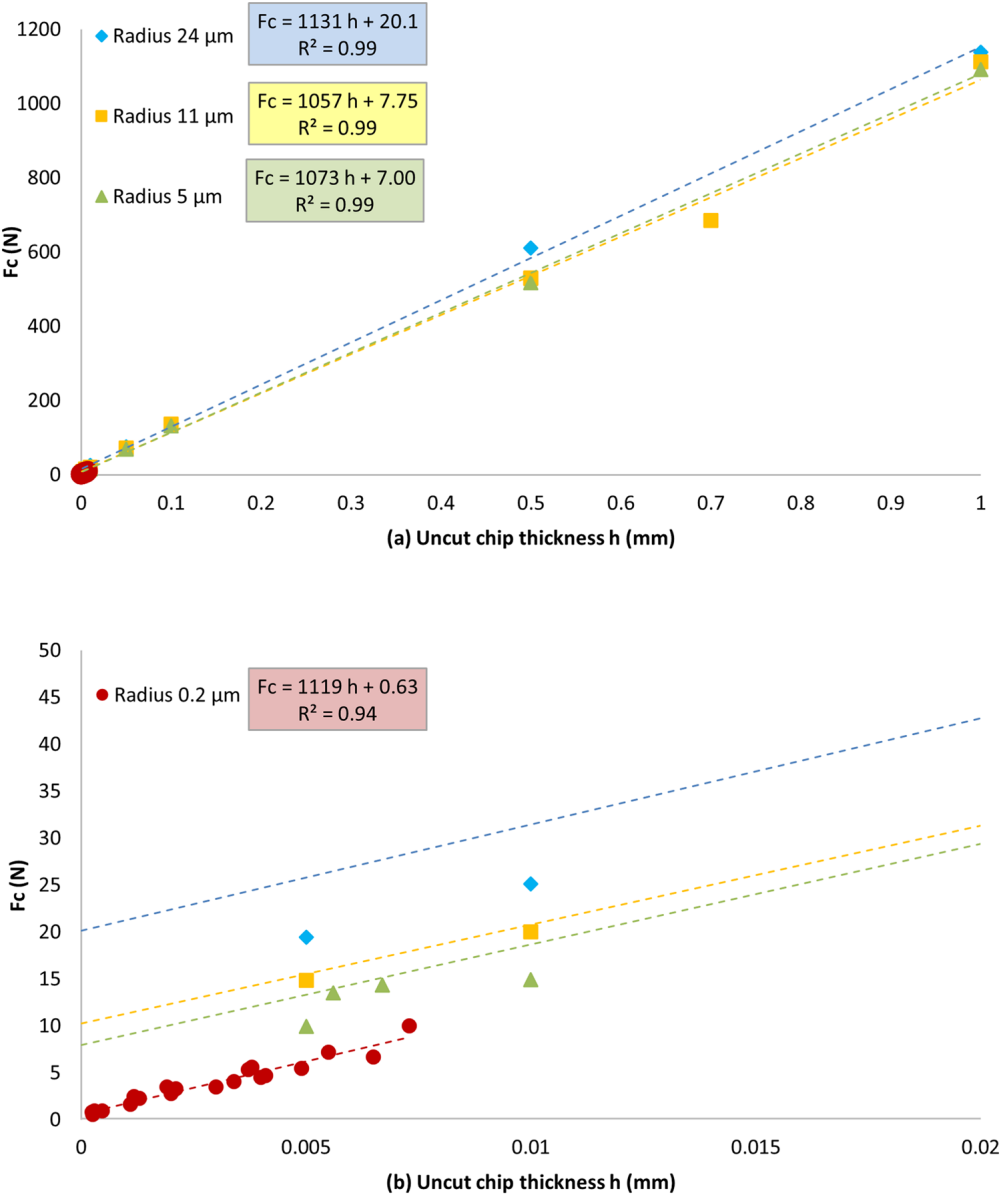
(**a**) Normalized cutting forces (Fc) depending on the uncut chip thickness for different tool tip radii. (**b**) A detail of the small feed values (dashed rectangle) is depicted in the bottom image.

Figure 5 shows a dependence of the normalized cutting force vs *h* for different tool tip radius for both micro and macro- experiments. For every tip radius the dependence is well approximated by a straight line. The coefficients of all linear fits (having the meaning of specific cutting energy) are very close to each other indicating close values of specific cutting energies in a broad range of tip radii. At the same time, the intersect terms of the fits differ by more than one order of magnitude for the smallest and largest tip radii, which is considered a signature of the size effect^26^. Moreover, as can be seen from Fig. 5b, all tool radii experimental points are systematically lower than the corresponding linear fit lines at small *h* values, also indicating the dependence of the cutting energy on *h*.

## Discussion

### Versatility of in-SEM micro-cutting system and first observations

Previously, Heo et al. proposed a linear cutting in-SEM device, realizing *h* in the range of tens of microns^12^ and force measurements in the range of tens of Newtons, yet the working principle of the force sensing has been completely omitted making it impossible to verify. Much greater precision was achieved by Fang et al. with *h* sizes down to 10 nm^13^. In the current work, an in-SEM micro-cutting setup has been implemented with the function of measuring the cutting force by image monitoring. The device is constituted of simple and commercially available elements, as presented in Fig. 1, and can provide values of h down to 200 nm. Such a simple construction defines device versatility in terms of:

- low cost commercial availability of the elements and flexibility in elements arrangement in an SEM chamber, which allows utilization of the setup for various types of materials, various workpiece geometries and different kinds of experiments;
- displacement and force measurements performed by image analysis in the area of the tool tip, which eliminates the necessity for extreme stiffness and precision of the rest of the construction;
- possibility for in-operando observation of the cutting process including chip generation leading to a detailed view of the different stages of deformation;
- possibility for a direct link of observed structural features and cutting peculiarities to the force acting between tool and the workpiece;
- possibility for a-posteriori analysis of the internal structure of the machined surface, the chip and the tool using various capabilities of the electron/ion microscope in the inert vacuum environment without bringing the sample to the air.

While these individual aspects represent certain technological innovation with respect to previous works, the possibility to link mechanical and microstructural aspects in one experiment by the proposed setup represents, in the opinion of the authors, a much more significant advantage.

In-operando microscopic observation of the cutting process allowed to approach metal cutting from a fundamental perspective. It can be confirmed that the cutting process in the nanometre regime is fundamentally similar to that at the macroscopic scale. Earlier^19,27^ it has been hypothesised that a chip generation is determined by the relation between the uncut chip thickness *h* and the tool edge radius rather than by *h* itself, however, this has been hardly confirmed experimentally as a sub-micron tool tip radius has been difficult to achieve in macroscopic machining. The in-SEM setup utilized in the present study allows to perform cutting within the micrometre range for *h,* yet keeping the cutting-edge radius smaller than *h*. In this process a chip generation identical to the macroscopic cutting is observed with all classical deformation regions present, which confirms the above hypothesis. The typical lamellae texture of the chips characteristic for macroscopic cutting is also present in microscopic cutting as shown in Fig. 2b, and the fraction of material in frictional contact with the tool depicts a microstructure refined by dynamical recrystallisation as observed macroscopically^3,28^. It is remarkable that this zone in principle exists in micro-cutting experiments, as the cutting speed is a few orders of magnitude lower than in macroscopic case and the friction should not cause a substantial heating necessary for recrystallization. The observation of classical deformation structure in microscopic cutting corroborates its identical principles to macro cutting and thus supports the feasibility of utilization of in-SEM micro-cutting to study fundamental microscopic features of the machining process.

A few more observations are here outlined: Thus, it has been directly shown that the thickness of the deformed layer in TSZ changes from one grain of the workpiece to another, as represented on Fig. 3b. As the difference between grains is only in their crystal lattice orientation to the surface, this effect can be attributed to the mechanical anisotropy of cubic lattices. It is known for pure aluminium, that the flow stress in the stronger crystal direction may double that found in a weaker direction^29^. Hence, the slip direction perpendicular to the surface can be favoured or not for strain propagation depending on the crystallographic orientation of the grain under cutting. Though such dependence may sound obvious, it has not been observed experimentally so far and would be difficult to observed if the tool radius exceeds the grain size, which is the typical case for macroscopic cutting.

Besides relating force dependencies to the microstructural processes, force measurement in micro-cutting opens up the possibility of extending the study of energetics of cutting to extremely small dimensions, and thus to reveal individual phenomena contributing to the cutting energy. This will be discussed in the following chapter. For this discussion the cutting forces measured in the steady state will solely be used.

### Evaluation of the multiscale cutting force data

As it was pointed out above, the possibility of measuring the cutting force during micro-cutting gives a unique opportunity to study the energetics of the cutting process on a scale range reduced by a few orders of magnitude and thus to reveal peculiarities passed unnoticed in a common macroscopic study.

Experimental data on cutting forces obtained in this work and shown in Fig. 5 is in a good agreement with previously published data^30,31^. In all these works a linear increase of cutting force with increase of *h* has been observed; however, at *h* below the tool tip radius the nonlinear variation of the cutting force was noted. Moreover, a vertical offset of the force curves depending on the tool radius has been observed^26^, similar to what is shown in Fig. 5.

Along with individual machining parameters like cutting forces, tool wear, material removal rate, temperature or chip ratio, an integral measure of the efficiency of machining, called specific cutting energy (also known as specific cutting force), was assigned as a reliable way to quantitatively evaluate machining^32^. Specific cutting energy (here after denoted as *K*_*c*_ after Drucker^33^) is defined as the energy required to machine a unit volume of material and can be expressed through the common parameters of orthogonal cutting as (where *E*_*c*_ and *V*_*c*_ are the cutting energy and cut volume respectively):

1 $$E_{c} = f_{c} \cdot d;\;\;\;V_{c} = h \cdot w \cdot d;\;\;\;K_{c} = \frac{{E_{c} }}{{V_{c} }} = \frac{{f_{c} }}{h \cdot w}$$

Here *f*_*c*_ is the cutting force, *h* is the uncut chip thickness, *w* is the width of cut and *d* is the length of cut. Note that *Kc* has a dimension of pressure and can be interpreted as an internal pressure of the material, which the tool has to overcome in order to cut.

At maximum *h*, the values of the cutting energy *K*_*c*_ for Alu7475 obtained as the slope of the linear fits on Fig. 5 are very close to 1100 MPa at all tip radii and correspond well to available literature data for a similar material (see Table 1).

**Table 1:** Comparison of the values of the Kc.

Material	Cutting speed	K_c_	Reference
Alu7475	0.5–500 mm/min	1100 MPa	Current work
Alu7075-T6	10–150 m/min	1300 MPa	Ng et al.^30^
Alu7075-T6	200–500 m/min	1000 MPa	Fang and Wu^31^
Alu7075-T6	525–1585 m/min	800 MPa	Rao and Shin^34^

The increase of the cutting energy at low h (also referred to as size effect in the cutting energy), though being noted, has not obtained a unanimous explanation. While e.g. Woon and Rahman^27^ have argued that this effect has purely geometric nature, i.e. is solely determined by the ratio between tool radius and h, Weber et al.^35^ have suggested that a material hardening should be a reason for variation of cutting energy with size. In this case, the size effect due to strain hardening has been related to much higher strain gradients and thus to higher dislocation densities at small tool radii. Fang et al.^36^ have also suggested an extreme case of nanocutting at the feeds smaller than inter dislocation distance (tens of nanometres), when the hardening is caused by the starvation of strain carriers. We will not consider this last mechanism as this scale is far beyond the reach of the proposed microcutting setup. In the following, utilizing the force measurements from both micro- and macroscale cutting experiments, we will try to discriminate the factors determining size dependence of the specific cutting energy.

**Figure 6:**
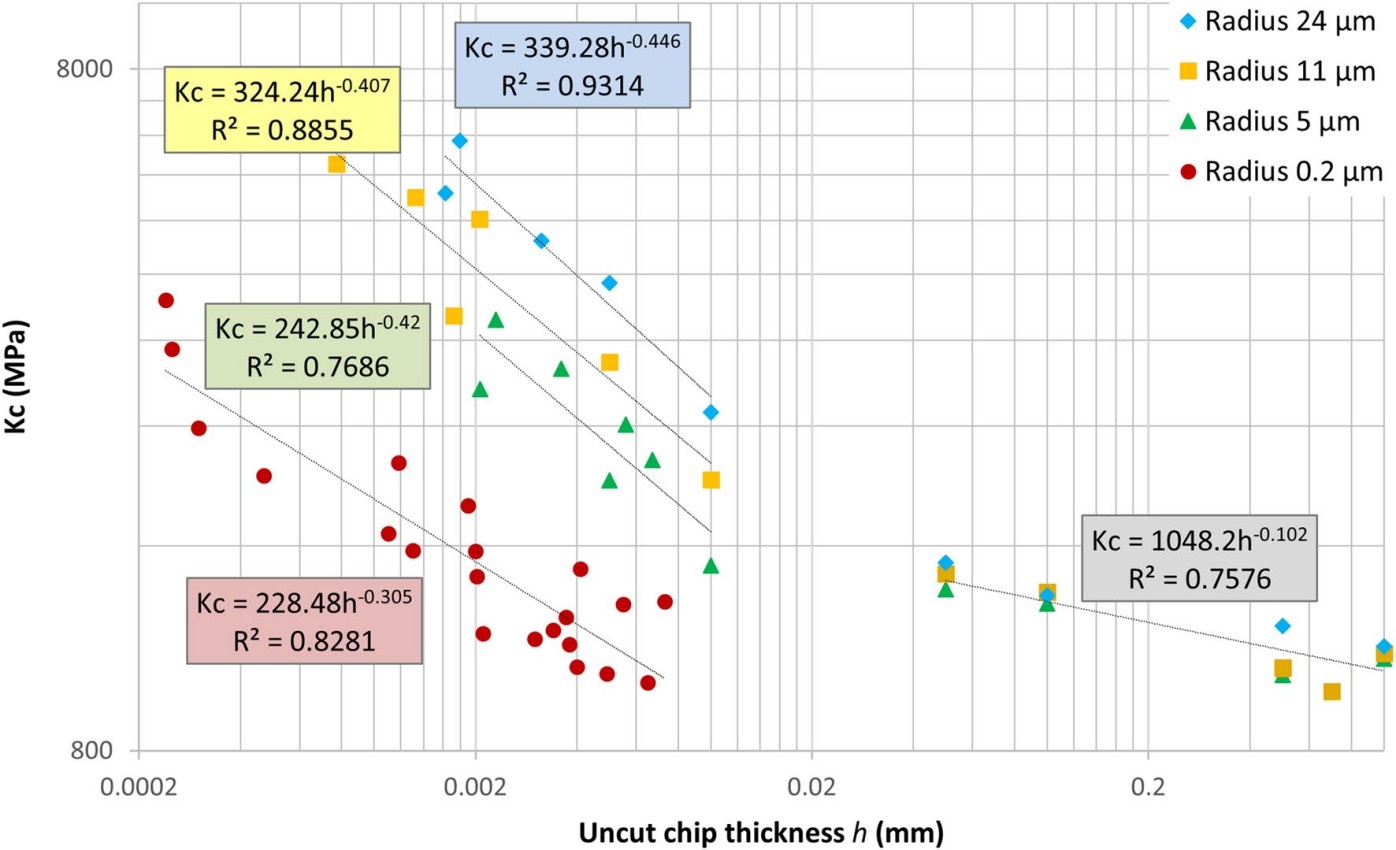
Plot of the measured specific cutting energy (Kc) depending on the uncut chip thickness (h).

Figure 6 plots the values of *K*_*c*_ calculated from the force measurements on Fig. 5 in log–log scale. Two features are immediately evident from this plot. First, the data can be well fitted by power laws, however, the exponent (slope of the lines) is different for feeds below 10 µm and above 50 µm. Such a change in the power law exponent is typically related to a fundamental alteration of a material property or to a change in the mechanism of the process (e.g. change of the plastic deformation mechanism in strain/stress curves). So, it can be concluded that at high and low feeds the processes determining the specific cutting energy are different. For very high feeds the power law exponent tends to zero corresponding to constant K_c_ equal to K_0_. At small *h* exponent is approaching -0.5 in power law, which corresponds to the friction contribution as will be discussed later. The second feature on Fig. 6 is that at low *h* the *K*_*c*_ depends on the tool radius, while above 50 µm the data for all tool radii coincide, as is expected at large RTS^17^.

### Modelling framework

Two main hypotheses have been used so far to explain the size effect in the specific cutting energy. As mentioned above, one is based on the contribution of the geometry of the tool (RTS) and friction, and the other refers to material hardening due to the shear strain occurring in PSZ. In this framework, let us consider both effects as individual components of the overall specific cutting energy. Hence, the total *K*_*c*_ can be expressed as:

2 $$K_{c} = K_{0} + K_{fric} + K_{PSZ}$$

“Methods” section gives a detailed expansion of this expression resulting in an Eq. (3):

3 $$K_{c} = \left\{ {\begin{array}{*{20}c} {\left( {1 + \mu \cdot \frac{R}{h}} \right) \cdot \left( {K_{0} + M\alpha Gb\sqrt {\frac{1/b}{{h \cdot \cos \theta + 0.5 \cdot R \cdot \left( {\sin \theta + \cos \theta - 1} \right)}}} } \right),} & { h \ge R} \\ {\left( {1 + \mu \cdot \sqrt {2 \cdot \frac{R}{h} - 1} } \right) \cdot \left( {K_{0} + M\alpha Gb\sqrt {\frac{1/b}{{h \cdot \cos \theta + 0.5 \cdot R \cdot \left( {\sin \theta + \cos \theta - 1} \right)}}} } \right),} & {h \le R} \\ \end{array} } \right.$$

where *μ* is a friction coefficient; *R* is a tool radius; *b* is the Burgers vector, *G* is the material’s shear modulus; *α* is a conversion factor of 0.5 as defined by Nix and Gao^37^; *M* is a parameter relating the flow stress to the shear modulus, for which a value of √3 is assumed according to von Mises yield criterion^38^; *ρ*_*PSZ*_ is a dislocation density, *b* is the step size of one dislocation and *θ* is a PSZ angle. Here the first bracket is related to the geometrical aspects of tool/workpiece interaction, in particular the second term describes the friction at the tool edge. It is notable that this term depends only on RTS, see further discussion. The second bracket is related to the properties of the material under cutting, in particular the second term describes the addition to the cutting energy due to the strain hardening in PSZ.

A few simple consequences can be directly derived from Eq. (3). Thus, at very large feeds both second terms tend to 0 and *K*_*c*_ becomes constant and equal *K*_*0*_. For the large tool radii *R* independently of *h* the strain hardening term is small and thus the size dependence of *K*_*c*_ is described by geometrical factors only (according to RTS concept). Only when both *h* and *R* are small, may the strain hardening term become significant and will start to contribute to the cutting energy.

**Figure 7:**
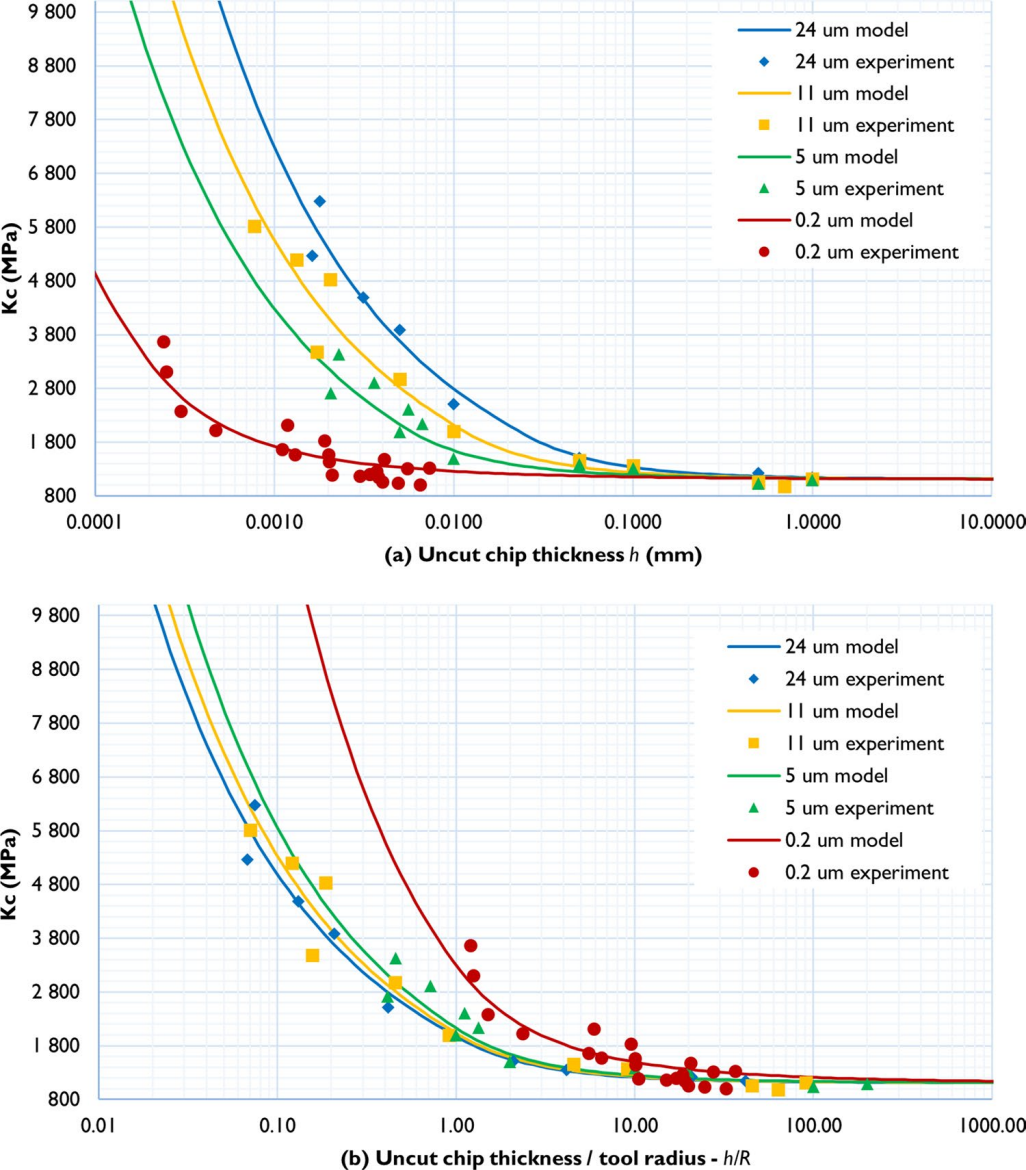
Analytical model (solid lines) and experimental data (points) for all tool edge radii: (**a**) specific cutting energy vs feed; (**b**) specific cutting energy vs RTS.

Verification of this analytical model against the data obtained in macro- and micro-cutting experiments is shown in Fig. 7. Two model parameters *K*_*0*_ and *μ* were fitted by minimising the mean square deviation simultaneously over the complete dataset. The resulting optimised value of *K*_*0*_ = 1.1 GPa is very close to the values determined from Fig. 5. The friction coefficient, μ = 0.65, obtained from fitting, agrees with the range of literature values as is seen from Table 2.

**Table 2:** Comparison of the values of the friction coefficient.

Material	Cutting speed	Friction coefficient	Reference
Alu7475	0.5–500 mm/min	0.65	Current work
Alu7075-T6	10–150 m/min	0.4–0.5	Ng et al.^30^
Alu 324.0	20–100 m/min	0.8–1	Faverjon et al.^39^
Alu alloy AK6	60–354 m/min	0.4–0.5	Stakhniv et al.^40^

Assuming the scattering of the experimental data, the model gives an adequate description in the whole range of the tool radii and *h* covered in experiments. On the one hand, this proves the validity of the proposed description, and on other hand, provides a method for the independent measurement of the friction coefficient and the specific cutting energy directly from the cutting experiments. Thus, the friction coefficient can be extracted from the slope of the curve at *h* smaller than the tool radius (independently of this radius), while the specific cutting energy corresponds to the asymptote of the curve at a value of *h* at least 10 times larger than the tool radius.

Figure 7b plots *K*_*c*_ vs *RTS*, separating the contributions coming from tool edge geometry and material hardening. In these coordinates the hardening contribution corresponds to the vertical separation of the curves—if the hardening did not take place (strain hardening term in Eq. (3) was equal to 0) the curves for all tool radii would join in one, as there would be only dependence of *K*_*c*_ on *R/h*, but not on *R*. It can be seen both from experimental points and from the model, that strain hardening contribution is present, but is minor, in the range of tool radii conventionally used in machining, and only becomes pronounced for sub-micrometre tool radii.

The proposed model does not arbitrarily include such cutting parameter as cutting velocity, though it is intrinsically included as the potential velocity dependence of the material properties: friction coefficient and shear modulus. The fact that the model is able to describe the cutting process in the micro- and macro-cutting experiments (3 orders of magnitude range in cutting speed and 2 orders in sizes) with the same values for material properties indicate that these properties do not significantly change in these ranges. Moreover, they can be measured directly in cutting conditions by a series of experiments with varying *h* (coefficient of friction and *K*_*0*_) or varying tool radius (shear modulus). It should be noted that only the measurement of cutting force is necessary to measure the friction by this approach, while the procedure used e.g. by Stakhniv et al. required both cutting and feed force measurement to compute the friction in the flank side^40^.

In summary, an in-SEM linear micro-cutting device has been designed and implemented by a simple arrangement of commercially available elements except for the cutting tool itself. A unique feature of the proposed device is its ability to provide cutting force measurements while the workpiece is fully accessible for SEM/FIB inspections. Proof of concept cutting experiments with *h* between 200 nm and 7 µm have been successfully performed.

The system permits the access to the chip generation details in the close contact between the tool and the workpiece. In-operando observation of this area lead to the following findings:

- All classical deformation regions (PSZ, SSZ and TSZ) are observed in micro-cutting experiments very similar to the macroscale. In particular, the SSZ observed in micro-cutting experiments has notably different structure to the rest of the chip (sign of dynamical recrystallization), despite a very small uncut chip thickness (*h*) and small cutting velocity. This confirms that the tool-workpiece interaction in microscale experiments is principally the same as in macroscale cutting, and thus the method can be used for studying industrially relevant processes.
- For the TSZ the dependence of its thickness from the crystallographic orientation of the grains has been clearly demonstrated. As TSZ is an affected layer residing on the final cut surface, it determines the consumer properties of the final piece and dependence of these properties on underlaying crystals orientation may be of practical relevance.

To validate the veracity of micro-cutting force measurements the data was compared to macro-cutting tests with varying tool edge radius performed in the same material on a linear cutting bench.

- It was shown that micro and macro cutting force measurements result in similar, yet systematically changing values of specific cutting energy for Alu7475. This confirmed the dependence of the specific cutting energy on the tool radius, known as “size effect”.
- This dependency for all sets of experimental data covering two orders of magnitude of tool sizes and three orders of magnitude of cutting velocity was described by a single analytical model. In this model the friction coefficient and the specific cutting energy were the only two fitting parameters. These values obtained by fitting of a complete dataset are in a very good agreement with existing literature data.
- The proposed model discriminates three contributions to the specific cutting energy and defines the conditions under which one or the other becomes significant, namely: the geometry and resultant friction constitutes the major contribution to the cutting energy at *h* below the tool radii for any tool edge radius; material hardening has minor contribution at the scale of industrial tools and only becomes notable for a sub-micron sharp cutting edges; at *h* exceeding the tool radius by 50 times and more the specific cutting energy of a material is well described by a constant.

Thus, the development of the present device has not only provided sub-micron in-operando access to the chip generation process and material structure analysis, it has also presented the necessary experimental data to validate a model for the specific cutting energy valid along several orders of magnitude of *h* and tool radius.

## Methods

### Building an in-SEM linear cutting setup with the force measurement system

The design of the cutting device inside an SEM is determined by the method of force measurement. The foundation of the concept is built upon the Kleindiek STFMA spring-table system in combination with a Kleindiek MM3A-EM micromanipulator fitted with a custom-made cutting micro-tool. The manipulator provides two rotational and one radial displacement axes. Rotational axes are used for tool positioning, feeding and retracting. The longitudinal displacement axis is used to advance the cutting tool against the workpiece. The design of the spring table in the form of double flat parallel spring limits possible movements of the spring holder to a linear motion parallel to the holder surface. By design this direction coincides with the cutting direction. Kleindiek Nanotechnik GmbH provided the elastic constant of the spring, 1059 ± 53 N/m (though other springs are available from the provider).

The cutting force was measured and plotted during machining by tracking of the workpiece displacement on quick stop SEM images on-line by a software developed in collaboration with Kleindiek Nanotechnik GmbH (see below).

The device was mounted on the sample stage inside an FEI Nanolab Helios 600 DualBeam FIB/SEM instrument (FEI, Netherlands) as in Fig. 1c. Both electron and ion columns were used for monitoring purposes, leading to two different points of view of the cut. Incorporation of the device into FIB/SEM instruments has additional advantages, thus the tool can be fabricated/sharpened by FIB milling in-situ directly before experiments if necessary; the cross-sections of the workpiece and the chip can be made and studied directly after experiments without exposition of the workpiece to the atmosphere. In the current work 5 and 10 keV electron and 30 keV Ga^+^ ion beams were used.

The exact measurements of feed (in terms of uncut chip thickness) and cutting width were made a posteriori from SEM images by measuring the geometry of the cutting path resulted from machining. For convenience, cutting direction was aligned with the tilt axis of the microscope, leading to an observation of the cutting process perpendicular to the cutting direction (as the view direction on Fig. 1a,b).

**Figure 8:**
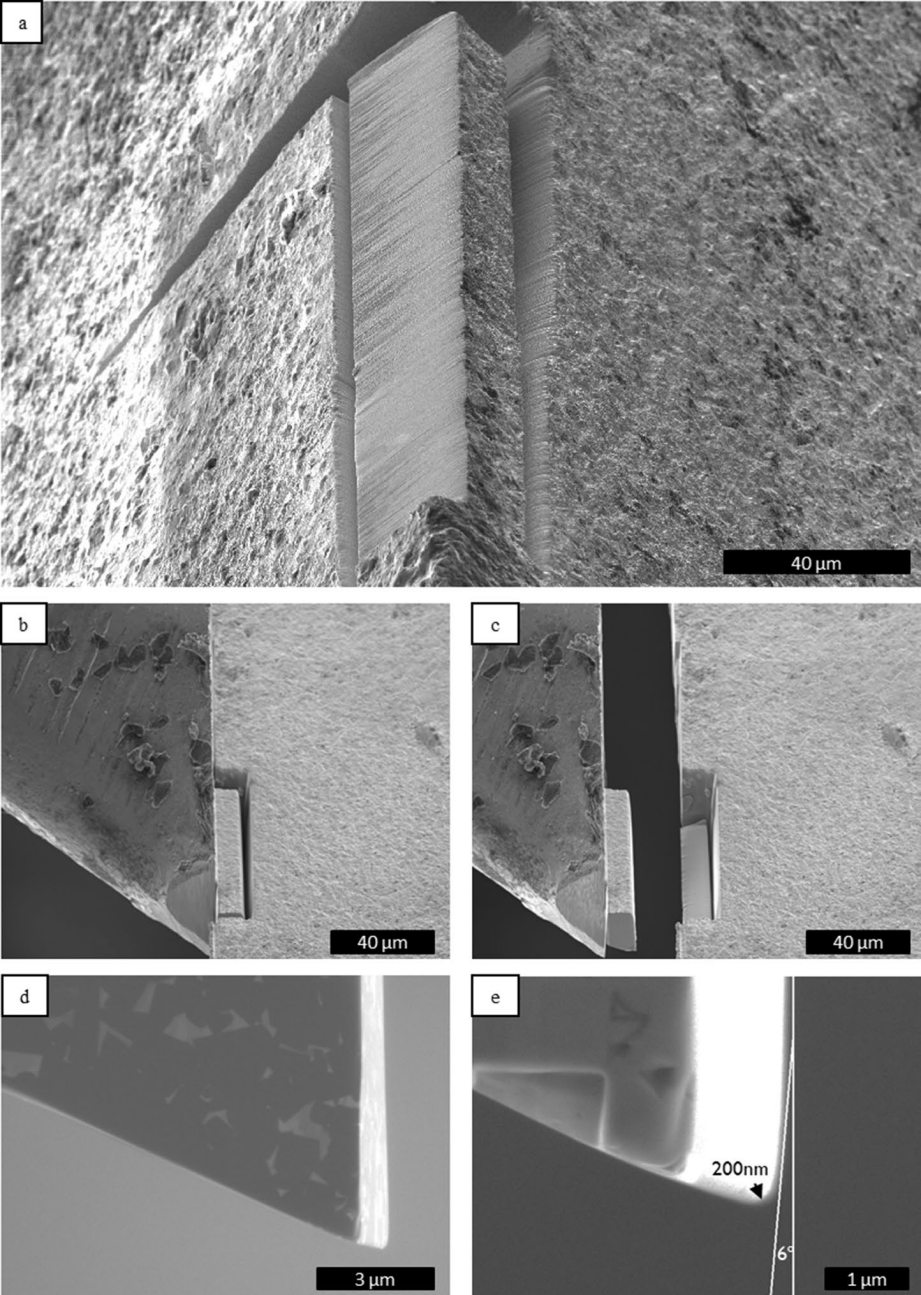
(**a**) Block of a WC–Co specimen milled with an ion beam. (**b**) Engagement of the block to the tool holder for welding by Pt deposition. (**c**) Extraction of the tool for further shaping operations. (**d**) Cutting tool after sharpening and (**e**) detail of the cutting edge of 200 nm radius and the angle produced by the ion de-focusing.

The cutting tool was fabricated from the same WC–Co H13A material as used for macroscopic cutting. The piece of material was placed in a FIB microscope and machined by ion beam as shown on the Fig. 8. Initially, a block of 80 × 30x10 µm was cut by FIB at high currents, between 9 and 60 nA. The block was cut all around leaving one side connected to the sample, and an aluminium arm of the micromanipulator approached the block. Then the block was welded to the aluminium holder by deposition of platinum at the interface via ion beam induced deposition using the gas injection system of the microscope. After this, the final cut was made to completely detach the tool from the macroscopic piece. In order to give the tool a proper surface finish and edge angle, the block was shaped by the ion beam at lower currents between 1 and 9 nA. By this last procedure, a cutting-edge radius of 200 nm was obtained (carbide grain size was 1 µm), a relief angle of 40° and a mean rake angle of − 6° in the proximity of the edge were obtained. Such negative angle is a consequence of the progressive de-focusing of the ion beam when sharpening the cutting edge from the bottom, a difficult to avoid effect when performing ion milling in the order of tens of microns which lead to a light curvature at the very edge even with low milling power. It should be noted that the fabrication of the cutting tool by FIB is a time-consuming operation, the time is required for milling the relatively large WC–Co block, and also for welding by ion induced platinum deposition. Yet, the tool can be reused multiple times (e.g. all systematic force measurements present in the current work were made from one and the same tool), and cleaning and re-sharpening by FIB can be performed as necessary.

### Micro-cutting tests

The experiments were made in annealed and rolled Aluminium 7475. As received the material had a grain size of 10–20 µm. ~ 20 cuts were made with a tool built as in Fig. 8, and a cutting tool/piece geometry as described in Table 3. A cutting speed of approximately 10 µm/s (0.6 mm/min) was used, with quick stops for imaging. The input parameters in the cutting experiments were the cutting speed, the width of cut and the geometry of the cutting tool. Since the exact feed size could not be setup by the current design of the device, it has been measured from SEM images after each experiment, and thus the uncut chip thickness values are not evenly distributed between 0.2 and 7 µm. The experimental plan is included in Table 3.

**Table 3:** Microscale experimental plan.

Machine-tool	Type	In-situ SEM cutting
Cutting tool	Insert tool reference	FIB cut from TPUN 160,308 TTM insert
	Coating	None
	Rake angle [°]	− 6 (± 2)
	Relief angle [°]	40 (± 2)
	Cutting edge radius (R) [µm]	0.2 (± 0.01)
Lubrication	Type	Dry
Workpiece	Material	Aluminium 7475
	Condition	Particle precipitation and mechanical forming
Cutting conditions	Operation	Linear cutting
	Cutting speed (v) [mm/min]	0.6
	*Width* [µm]	11–23
	Uncut chip thickness (h) [mm]	0.0002–0.007

After cutting experiments, chips and workpieces were examined for width and uncut chip thickness parameters determination and their cross-sections were prepared for the structure analysis.

### Macroscopic experimental set-up

**Figure 9:**
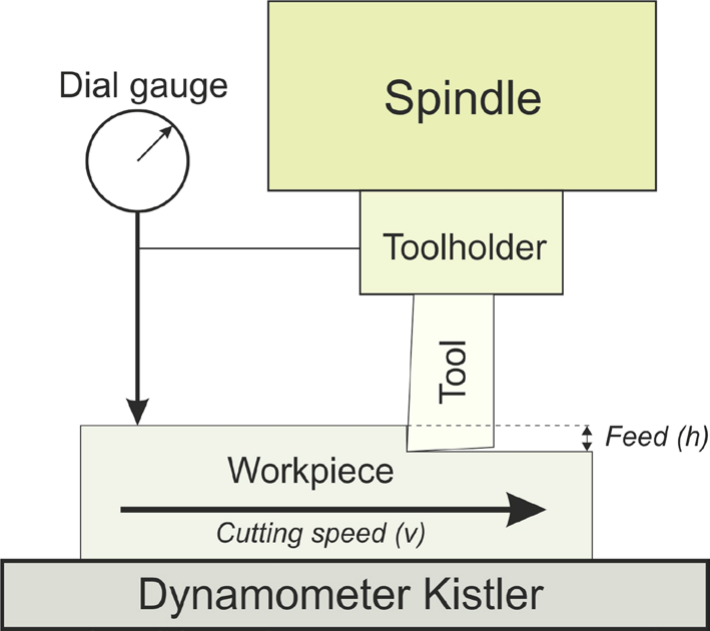
Sketch of the macroscale cutting test setup.

Macroscopic metal cutting experiments were carried out ex-situ on industrial equipment with commercially available tools. A *Lagun* machining centre with computer numerical control (CNC) from Fagor, model 8070 was used. For cutting force monitoring samples were mounted on a dynamometer Kistler 9129AA. The setup included a high precision dial gauge fixed on the tool-holder that measured the real feed in-process to ensure that the lowest feeds were achieved (see Fig. 9).

**Figure 10:**
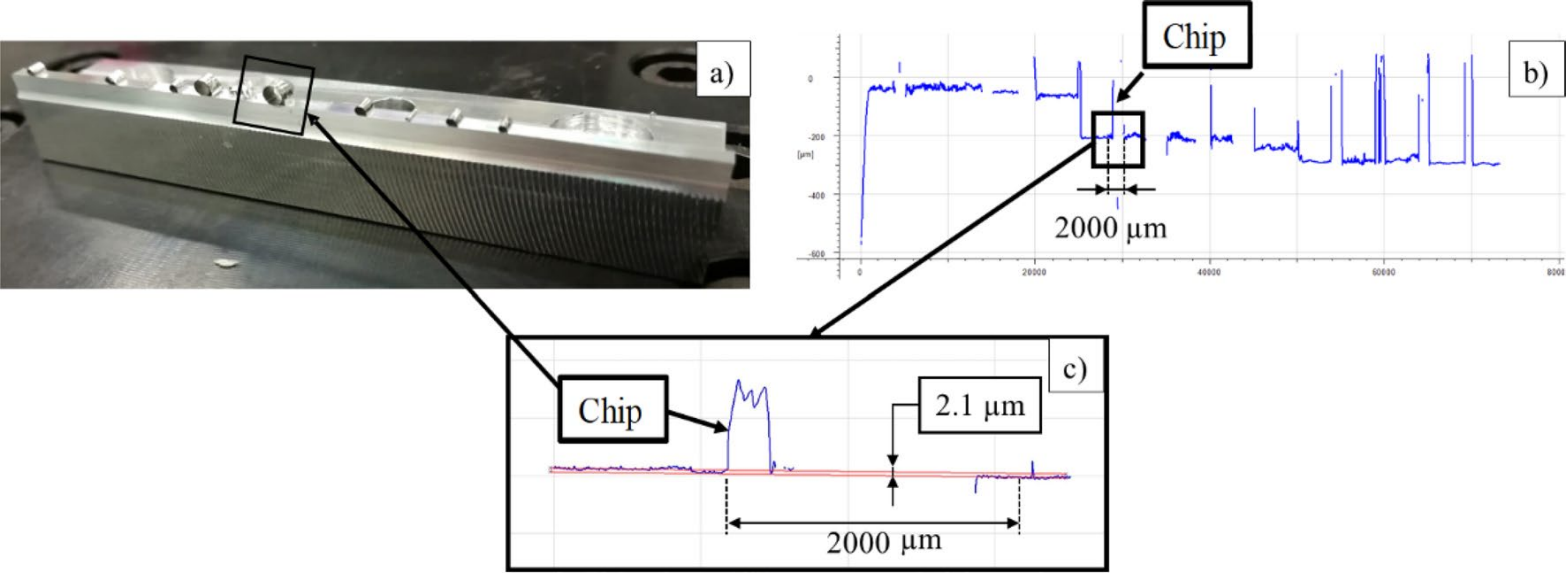
(**a**) A workpiece with multiple quick-stops, (**b**) overview profile obtained with the Alicona IFG4 at 20X and (**c**) detailed profile of the selected area at higher resolution (50X).

Nevertheless, for feeds smaller than 5 µm the machine vibration did not permit to use this methodology. Hence, an optical profilometer Alicona IFG4 was used to measure the real feed a posteriori. The methodology consisted of making quick-stops and then measuring the vertical distance between the machined and not machined surfaces by the optical device to obtain the real feed (see Fig. 10).

Macro-cutting experiments were made exactly in the same material as micro-cuts: annealed and rolled Aluminium 7475 from the same batch. A total of 20 cutting conditions were tested at 500 mm/min (the minimum stable speed in the equipment) with feeds between 1 and 0.001 mm. Three repetitions were done for each cutting condition leading to a total of 60 tests. The details and parameters of macroscopic cutting tests are listed in Table 4.

**Table 4:** Macroscale experimental plan.

Machine-tool	Type	High Speed Machining Centre Lagun
Cutting tool	Insert tool reference	TPUN 160308 TTM—uncoated
	Coating	None
	Rake angle [°]	6 (± 0.5)
	Relief angle [°]	5 (± 0.5)
	Cutting edge radius (R) [µm]	5, 11, 24 (± 1)
Lubrication	Type	Dry
Workpiece	Material	Aluminium 7475
	Condition	Particle precipitation and mechanical forming
Cutting conditions	Operation	Linear cutting
	Cutting speed (v) [mm/min]	500 (± 5)
	Width [mm]	2 (± 0.05)
	Uncut chip thickness (h) [mm]	0.001, 0.002, 0.005, 0.01, 0.05, 0.1, 0.5, 1 (± 5%)

### Model development

The model for *K*_*c*_ includes the contributions of the friction of the tool edge against the workpiece *K*_*fric*_, and of the increase of material strengthening in the PSZ *K*_*PSZ*_. The total *K*_*c*_ can be expressed as:

4 $$K_{c} = K_{0} + K_{fric} + K_{PSZ}$$

Here *K*_*0*_ is the ground constant cutting energy corresponding to the conditions at which cutting energy experimentally does not depend on sizes, as detailed in the discussion.

The friction force is considered to obey the Coulombs^41^ approach as in Eq. (5), where the normal force is calculated assuming *K*_*0*_ as a pressure and *p* the contact area (per unit width) projected normal to the workpiece surface (Eq. 6). Here we assume, that only the part of the tool pressing against an uncut material contributes to the normal force. As the rake angle is assumed to be 0, the friction of the chip against the tool does not have a force component in the direction of cutting and contributes only to the feed force, but not to the cutting force.

5 $$F_{fric} = \mu \cdot F_{N}$$

6 $$F_{N} = p \cdot K_{0}$$

**Figure 11:**
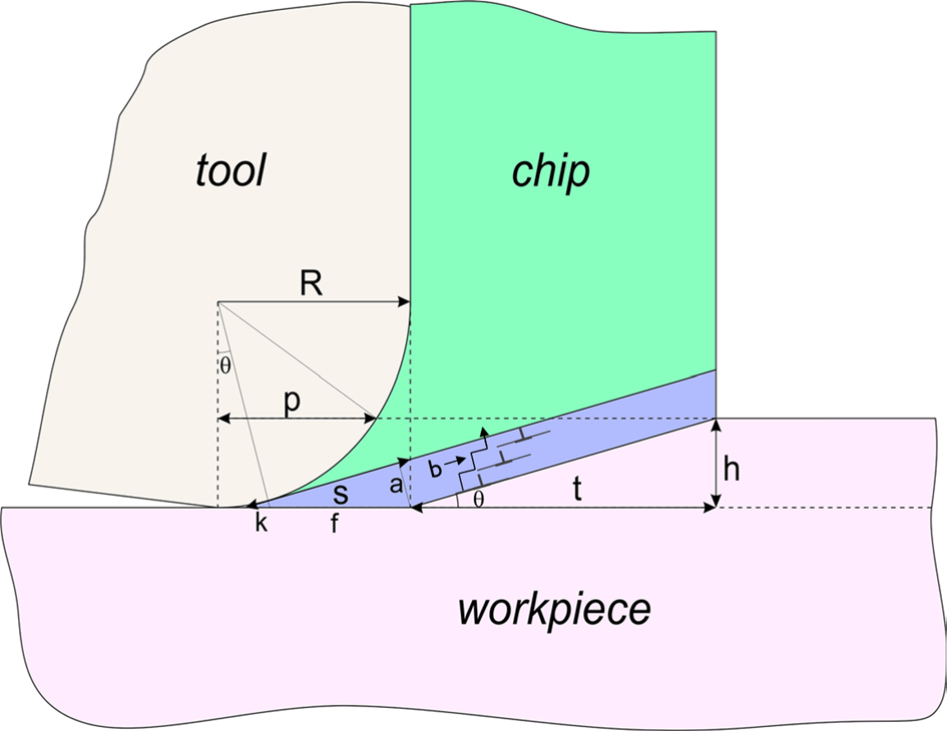
Sketch of dimensions defining the cutting geometry used in the calculations of K_fric_ and K_PSZ_.

In order to calculate *p*, two domains are considered. When the feed is larger than the radius, *p* is equivalent to the tool radius. For the smaller feeds it is calculated as (see also Fig. 11):

7 $$\begin{gathered} p^{2} + \left( {R - h} \right)^{2} = R^{2} \hfill \\ p^{2} + R^{2} + h^{2} - 2 \cdot R \cdot h = R^{2} \hfill \\ p^{2} = 2 \cdot R \cdot h - h^{2} \hfill \\ \end{gathered}$$

where *R* is the edge radius and *h* is the uncut chip thickness. Thus, the normal projection of the contact area *p* is described as:

8 $$p = \left\{ {\begin{array}{*{20}c} {R,} & {h \ge R} \\ {\sqrt {2 \cdot R \cdot h - h^{2} } ,} & {h \le R} \\ \end{array} } \right.$$

Then, the friction related contribution to the *K*_*c*_ can be written as:

9 $$K_{fric} = \frac{{F_{fric} }}{h} = \left\{ {\begin{array}{*{20}c} {\mu \cdot K_{0} \cdot \frac{R}{h},} & {h \ge R} \\ {\mu \cdot K_{0} \cdot \sqrt {2 \cdot \frac{R}{h} - 1} ,} & {h \le R} \\ \end{array} } \right.$$

Equation (9) points out that the friction force contribution is proportional to the edge curvature projected on the cutting direction and the friction coefficient. According to Fig. 11, this projection is constant for h larger than the edge radius, and variable with h in the opposite case.

The strengthening of the PSZ is considered to be the consequence of the increase of the dislocation density due to the fact, that the width of PSZ scales down with the tool tip radius while the induced strain in chip generation process remains the same.

Strain induced material strengthening contribution to the cutting energy is considered following the Taylor’s approach^42^:

10 $$K_{PSZ} = M \cdot \alpha \cdot G \cdot b\sqrt {\rho_{PSZ} }$$

11 $$\rho_{PSZ} = N/V_{PSZ}$$

12 $$N = s/b$$

where *G* is the material’s shear modulus, M is a parameter relating the flow stress to the shear modulus, for which √3 is assumed according to von Mises yield criterion^38^, *ρ*_*PSZ*_ is a dislocation density generated by the shear in the primary shear zone, *s* is the total shear and b is the step size of a singular dislocation. We should be cautious assuming a value of *α*, a conversion factor to describe the hardening sensitivity to dislocation density for metals. Values from 2 to 5 have been reported, being the lower values for annealed materials where the mean path distance of dislocations is relatively large (low dislocation interaction). The approach in this work assumes a very short path distance associated with an environment rich in dislocation sources. Similar to nanoindentation tests, *α* is fixed to 0.5 as by Nix and Gao^37^. The dislocation density *ρ*_*PSZ*_ can be estimated from the number of dislocations *N* necessary to accommodate shear *s* divided by the volume (Eq. 11). The number of dislocations is calculated as the number of lattice steps *b* (dislocation Burgers vector) needed to reach the total strain (Eq. 12).

The volume of deformation in the primary shear zone can be estimated following the geometry proposed by Asad^43^ from molecular dynamics calculations. That geometry is schematically outlined in Fig. 11, where the volume of the primary shear zone is described by a trapezium: the total area of the PSZ (volume per unitary width) can be approximated by the areas of a parallelogram and a triangle.

13 $$\begin{aligned} V_{PSZ} & = \left( {a \cdot \frac{t}{\cos \theta }} \right)_{paralleloram} + \left( {\frac{a \cdot s}{2}} \right)_{triangle} \\ V_{PSZ} & = a \cdot \left( {\frac{t}{\cos \theta } + \frac{s}{2}} \right) \\ \end{aligned}$$

Here *V*_*PSZ*_ is the volume of the PSZ layer per unit of width, *a* is the PSZ thickness, *t* is the chip thickness and *θ* is a PSZ angle defined as *θ* = *Arctan(h/t)*. The thickness *a* in the PSZ is defined as (see Fig. 11):

14 $$\begin{gathered} a = \left( {f + k} \right) \cdot \sin \theta = s \cdot \cos \theta \cdot \sin \theta \hfill \\ \tan \theta = \frac{h}{t};t \cdot \sin \theta = h \cdot \cos \theta \hfill \\ \end{gathered}$$

Then Eq. (11) can be written as:

15 $$\begin{gathered} \rho_{PSZ} \cdot b = \frac{s}{{V_{PSZ} }} = \frac{1}{{\cos \theta \cdot \sin \theta \cdot \left( {\frac{t}{\cos \theta } + \frac{s}{2}} \right)}} \hfill \\ \frac{1}{t \cdot \sin \theta + 0.5s \cdot \cos \theta \cdot \sin \theta } = \frac{1}{h \cdot \cos \theta + 0.5a} \hfill \\ \end{gathered}$$

Hence, Eq. (14) becomes:

16 $$\begin{gathered} a = \left( {f + k} \right) \cdot \sin \theta = \left( {R \cdot \left( {1 - \tan \theta } \right) + \frac{{\left( {\frac{R}{\cos \theta } - R} \right)}}{\sin \theta }} \right) \cdot \sin \theta \hfill \\ R \cdot \left( {\sin \theta - \sin \theta \cdot \frac{\sin \theta }{{\cos \theta }} + \frac{1}{\cos \theta } - 1} \right) = R \cdot \left( {\frac{1 - \sin \theta \cdot \sin \theta }{{\cos \theta }} + \sin \theta - 1} \right) \hfill \\ a = R \cdot \left( {\cos \theta + \sin \theta - 1} \right) \hfill \\ \end{gathered}$$

According to that, the density of dislocations in the primary shear zone can be computed as

17 $$\rho_{PSZ} = \frac{1}{{b \cdot \left( {h \cdot \cos \theta + 1/2 \cdot R \cdot \left( {\cos \theta + \sin \theta - 1} \right)} \right)}}$$

subsequently the contribution of the strain induced hardening in the PSZ is

18 $$K_{PSZ} = M\alpha Gb\sqrt {\frac{1/b}{{h \cdot \cos \theta + 0.5 \cdot R \cdot \left( {\sin \theta + \cos \theta - 1} \right)}}}$$

Here it should be noted that the expressions for the friction force and *K*_*fric*_ were derived in Eqs. (6), (8) assuming constant cutting energy *K*_*0*_, i.e. not accounting for the strain hardening. The contribution of the friction (Eq. 9) and strengthening (Eq. 18) can be inserted in Eq. (4) for constructing the general equation of the model (consider, that accounting for the strain hardening we have to substitute K_0_ for (K_0_ + K_PSZ_) in Eq. (9))):

19 $$K_{c} = \left\{ {\begin{array}{*{20}c} {\left( {1 + \mu \cdot \frac{R}{h}} \right) \cdot \left( {K_{0} + M\alpha Gb\sqrt {\frac{1/b}{{h \cdot \cos \theta + 0.5 \cdot R \cdot \left( {\sin \theta + \cos \theta - 1} \right)}}} } \right),} & {h \ge R} \\ {\left( {1 + \mu \cdot \sqrt {2 \cdot \frac{R}{h} - 1} } \right) \cdot \left( {K_{0} + M\alpha Gb\sqrt {\frac{1/b}{{h \cdot \cos \theta + 0.5 \cdot R \cdot \left( {\sin \theta + \cos \theta - 1} \right)}}} } \right),} & {h \le R} \\ \end{array} } \right.$$

The parameters used for the analytical calculations are summarized in the Table 5.

**Table 5:** Parameters for modelling Aluminium 7475.

Type	In-situ SEM cutting
Burgers vector (b)	2.86 × 10^–10^ m^44^
Shear modulus (G)	27.1 × 10^9^ Pa^45^
Taylor factor (α)	0.5^37^
Shear to normal stress factor (M)	$\sqrt 3$^37^
Specific cutting energy (K_0_)	1.1 GPa (fitted)
Friction coefficient (µ)	0.65 (fitted)

### Software design for the force measurement system

The software has been designed and implemented for on-line assistance of the experiments made in-SEM. The software monitors the directory (local or remote) where the images are stored during the experiment and processes the images on-line. It calculates the force based on the shift of workpiece held by a bending spring as shown in Fig. 1. The cutting force is directly proportional to the displacement of the base (the workpiece) as:

20 $$F_{c} = \, K \cdot x$$

where *F*_*c*_ is the cutting force, *K* is the spring constant (for all experiments in this work the spring with the constant of 1059 N/m has been used) and *x* is the displacement, as shown in Fig. 1. The displacement of the tool is measured as well and the difference between two gives the relative movement of the tool against the workpiece.

**Figure 12:**
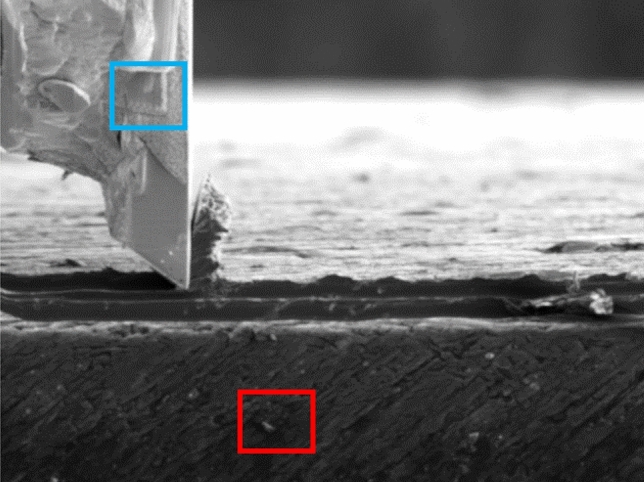
ROIs subject of tracking on the tool (blue) and the workpiece (red) for computing cutting force and displacement.

The tracking is performed using cross-correlation function^46^. In order to track the position of the tool and the workpiece, the software follows the positions of user defined regions of interest (ROI) one for each of the tool and the workpiece, as in Fig. 12. The tracking in successive images provides subpixel positioning of the tool and the workpiece on-line during experiment. A posteriori refinement of the positions can be done as well.

**Figure 13:**
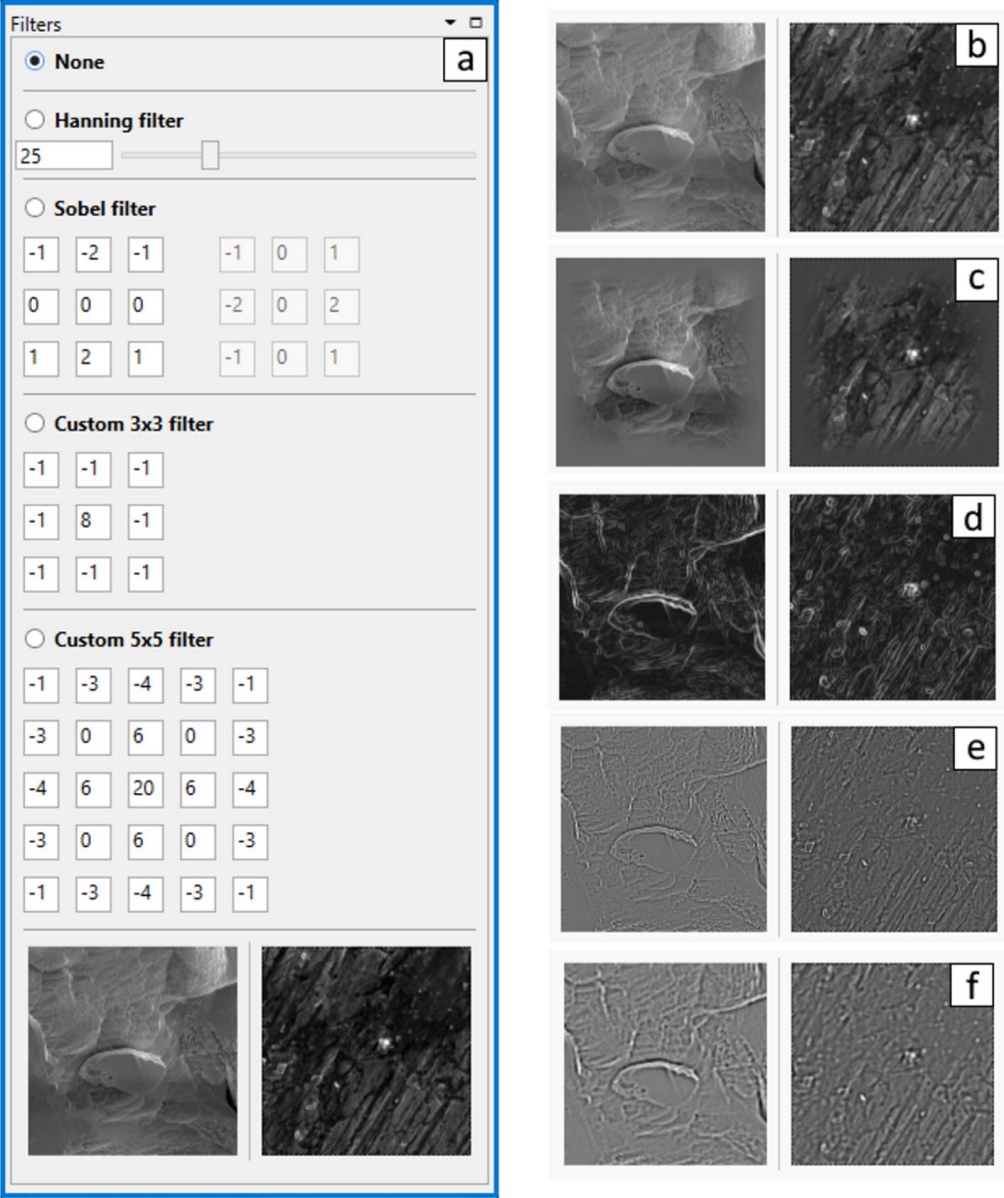
(**a**) Image filters panel; (**b**–**f**) ROIs on the cutting tool (left) and on the workpiece (right) processed by different filters—(**b**) without image filtering, (**c**) Hanning filter, (**d**) Sobel filter, (**e**) 3 × 3 kernel high pass filter, (**f**) 5 × 5 kernel high pass filter.

In order to improve the sharpness and unambiguity of cross-correlation maximum, several adaptive image filters are implemented. Figure 13a shows a screenshot of the filter page. The selector allows to apply in a SEM image (Fig. 13b) the *Hanning filter* to use the Hann function^47^ (Fig. 13c), the *Sobel filte*r to use the Sobel operator^48^ (Fig. 13d), or a custom defined 3 × 3 (Fig. 13e) or 5 × 5 (Fig. 13f) convolution matrix.

A successful tracking of the tool and workpiece displacements define the pairs of values for tool/workpiece movement and the force. This data can be plotted in various representations and monitored on-line: either force–displacement (Fig. 4) or validation of the raw data for each of the variables or any other combination of two parameters. Raw and calculated values can be saved in different formats for further processing in the other software. The following features are implemented as well: pixel size calibration—manual or from the tiff tags (currently for FEI microscopes only), viewing angle correction, constant drift correction, various options for image ordering, unlimited number of images, GUI customisation.
